# The complete chloroplast genome of the medicinal plant *Paederia foetida* L.

**DOI:** 10.1080/23802359.2022.2087563

**Published:** 2022-07-07

**Authors:** Wei Wang, Tao Xu, Xiangwen Song, Cunwu Chen, Dong Liu, Bangxing Han, Shanyong Yi

**Affiliations:** a Department of Biological and Pharmaceutical Engineering, West Anhui University, Luʼan, Anhui, P. R. China; b Anhui Engineering Laboratory for Conservation and Sustainable Utilization of Traditional Chinese Medicine Resources, West Anhui University, Luʼan, Anhui, P. R. China

**Keywords:** *Paederia foetida*, complete chloroplast genome, phylogenetic analysis

## Abstract

*Paederia foetida* L. belonging to Rubiaceae family is a perennial medicinal herb widely distributed in India and China. The first complete chloroplast genome sequence of *P. foetida* was assembled and characterized in this study. The total chloroplast genome was 153,591 bp in length with 37.74% GC content, composed of a large single-copy (LSC) region of 83,677 bp, a small single-copy (SSC) region of 16,888 bp and a pair of inverted repeat (IR) regions of 26,513 bp. The whole chloroplast genome encoded 133 genes, including 88 protein-coding genes, 37 tRNA genes and 8 rRNA genes. Phylogenetic analysis of 30 chloroplast genomes strongly suggested that *P. foetida* was closely related to *P. scandens.*

*Paederia foetida* L. (1767) belongs to Rubiaceae, which is an herbaceous perennial medicinal plant widely distributed in India and China (Kumar et al. 2015). The whole plant of *P. foetida* has medicinal value that is used as a folk medicine for treating rheumatism, diarrhea, inflammation, piles, dysentery, etc. (Upadhyaya 2013; Wang et al. 2014). Iridoid glycosides, saponins, phenols, flavonoids, and steroids are its important bioactive compounds (Upadhyaya 2013; Wang et al. 2014). However, there is no study about the complete chloroplast genome of *P. foetida*. In this study, the complete chloroplast genome sequence of *P. foetida* was established and characterized. The complete chloroplast of *P. foetida* will have significance for contributing to the research on its phylogenetic position in Rubiaceae.

The fresh leaves of *P. foetida* were collected from the medicinal botanical garden of of West Anhui University, Lu’an, Anhui Province, China (31°77′ N, 115°93′ E). The voucher specimen was deposited in the Herbarium of West Anhui University (voucher number WAU-JST-20220201-1, Wei Wang, 02000155@wxc.edu.cn). *P. foetida* is not a protected plant in China, and we collected it not from the private or protected area that required permission. The total genomic DNA of *P. foetida* was extracted according to the modified CTAB protocol (Doyle and Doyle 1987). The whole genome sequencing was performed using the Illumina Hiseq platform at Hefei Biodata Biotechnologies Inc. (Hefei, China). The complete chloroplast genome was filtered and assembled using program fastp (Chen et al. 2018) and SPAdes assembler 3.10.0 (Bankevich et al. 2012), respectively. This draft genome was annotated using GeSeq (Tillich et al. 2017) and BLASTx (Gish and States 1993).

The chloroplast genome of *P. foetida* was 153,591 bp in length (GenBank accession number: OL449949). The genome consisted of a large single-copy region (LSC, 83,677 bp), a small single-copy regions (SSC, 16,888 bp) and a pair of inverted repeat regions (IR, 26,513 bp). It contained 133 genes comprising 88 protein-coding genes, 37 tRNA genes, and 8 rRNA genes. Among them, nineteen of them contained two exons and four genes (*paf*I, *clp*P1 and *rps*12) contained three exons. The overall GC content in the *P. foetida* chloroplast genome was 37.74% with 35.63%, 31.96% and 42.92% for LSC, SSC and IR regions, respectively. Within the *P. foetida* chloroplast genome, seven protein-coding, eight tRNA and four rRNA genes were duplicated in IR regions.

The phylogenetic analysis was conducted based on the complete chloroplast genome (full DNA) sequences of *P. foetida* and 29 other related species and alignment was performed with MAFFT v7.307 (Katoh and Standley 2013). *Mitragyna speciosa* and *Neolamarckia cadamba* were used as out-groups. A maximum likelihood (ML) tree was constructed using the FastTree version 2.1.10 (Price et al. 2010). The phylogeny indicated that *P. foetida* was closely related to *P. scandens* (Figure 1). The complete cp genome sequence of *P. foetida* will provide useful information for phylogenetic and evolutionary studies in Rubiaceae.

**Figure 1.**
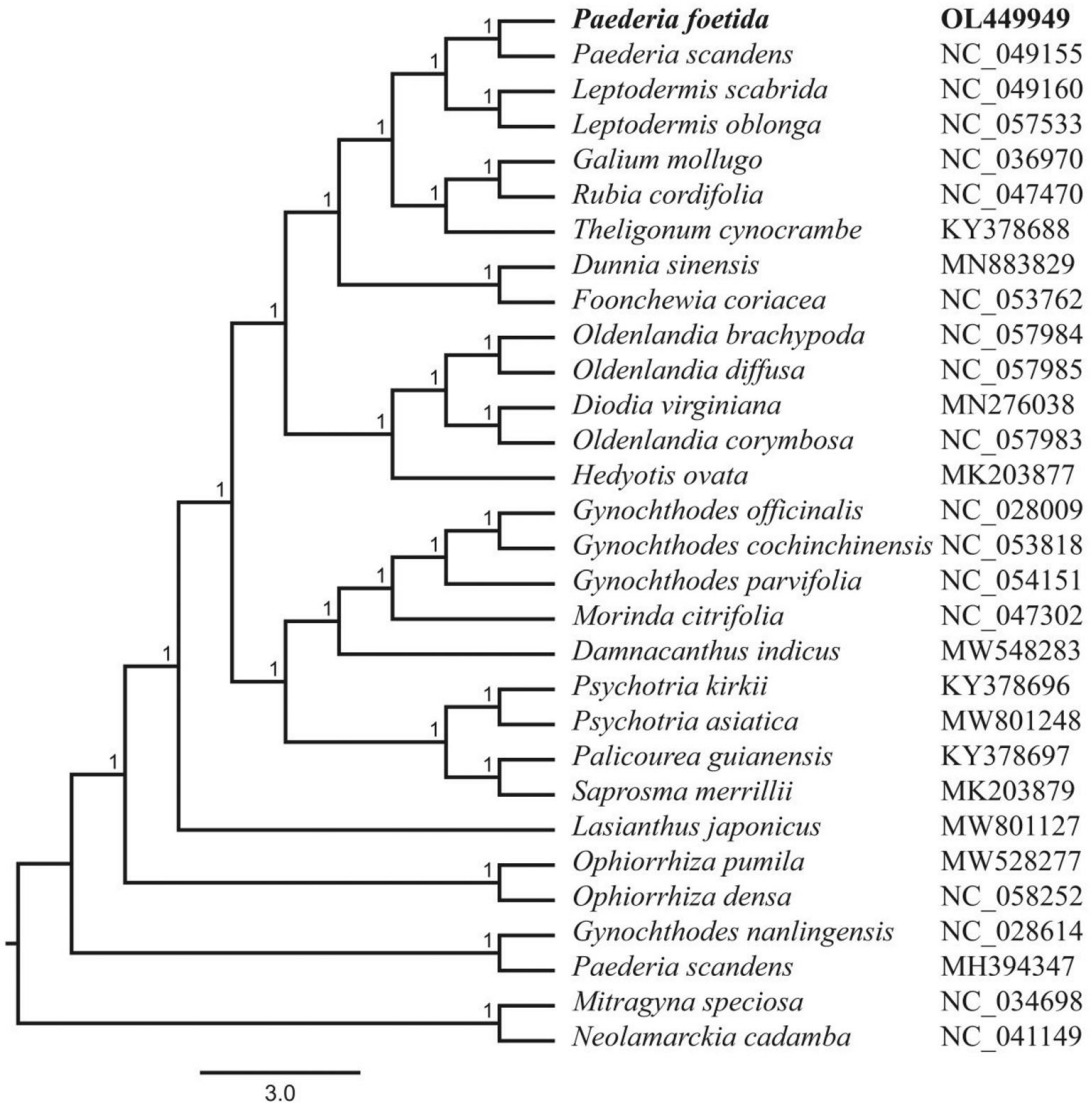
Maximum Likelihood phylogenetic tree based on complete chloroplast genomes of 30 species (*Mitragyna speciosa* and *Neolamarckia cadamba* were used as out-groups). A total of 1000 bootstrap replicates were computed and the bootstrap support values are shown at the branches.

## Author contributions

Conception and design: Yi S and Han B; data analysis and interpretation: Wang W, Xu T and Song X; the plant material collecting and identifying, manuscript writing and revising: Wang W, Yi S, Chen C and Liu D; All authors have read and approved the final manuscript and agree to be accountable for all aspects of the work.

## Data Availability

The genome sequence data of *P. foetida* that support the findings of this study are openly available in GenBank of NCBI at (https://www.ncbi.nlm.nih.gov/) under the accession no. OL449949. The associated BioProject, SRA, and Bio-Sample numbers are PRJNA782849, SRR17013866, and SAMN23402000, respectively.
